# Stabilization of *Picea abies* Spruce Bark Extracts within Ice-Templated Porous Dextran Hydrogels

**DOI:** 10.3390/polym16192834

**Published:** 2024-10-07

**Authors:** Roxana Petronela Damaschin, Maria Marinela Lazar, Claudiu-Augustin Ghiorghita, Ana Clara Aprotosoaie, Irina Volf, Maria Valentina Dinu

**Affiliations:** 1 “Cristofor Simionescu” Faculty of Chemical Engineering and Environmental Protection, “Gheorghe Asachi” Technical University of Iasi, Prof. Dimitrie Mangeron Boulevard 73, 700050 Iasi, Romania; roxanadamaschin.rd@gmail.com; 2 “Petru Poni” Institute of Macromolecular Chemistry, Grigore Ghica Voda Alley 41A, 700487 Iasi, Romania; maria.lazar@icmpp.ro (M.M.L.); claudiu.ghiorghita@icmpp.ro (C.-A.G.); 3 Faculty of Pharmacy, “Grigore T. Popa” University of Medicine and Pharmacy, Universitatii Street 16, 700115 Iasi, Romania; claraaprotosoaie@gmail.com

**Keywords:** antimicrobial, antioxidant, dextran, morphology, porous hydrogels, spruce bark extracts, stabilization approach

## Abstract

Porous hydrogels have brought more advantages than conventional hydrogels when used as chromatographic materials, controlled release vehicles for drugs and proteins, matrices for immobilization or separation of molecules and cells, or as scaffolds in tissue engineering. Polysaccharide-based porous hydrogels, in particular, can address challenges related to bioavailability, solubility, stability, and targeted delivery of natural antioxidant compounds. Their porous structure enables the facile encapsulation and controlled release of these compounds, enhancing their therapeutic effectiveness. In this context, in the present study, the cryogelation technique has been adopted to prepare novel dextran (Dx)-based porous hydrogels embedding polyphenol-rich natural extract from *Picea abies* spruce bark (SBE). The entrapment of the SBE within the Dx network was proved by FTIR, SEM, and energy-dispersive X-ray spectroscopy (EDX). SEM analysis showed that entrapment of SBE resulted in denser cryogels with smaller and more uniform pores. Swelling kinetics confirmed that higher concentrations of Dx, EGDGE, and SBE reduced water uptake. The release studies demonstrated the effective stabilization of SBE in the Dx-based cryogels, with minimal release irrespective of the approach selected for SBE incorporation, i.e., during synthesis (~3–4%) or post-synthesis (~15–16%). In addition, the encapsulation of SBE within the Dx network endowed the hydrogels with remarkable antioxidant and antimicrobial properties. These porous biomaterials could have broad applications in areas such as biomedical engineering, food preservation, and environmental protection, where stability, efficacy, and safety are paramount.

## 1. Introduction

The stabilization of natural organic compounds in polymer-based matrices for biomedical applications has emerged as a critical area of research, with a particular focus on natural polyphenols due to their remarkable biological properties. These compounds raise a significant interest both in the pharmaceutical field, as medicinal treatments, and in agriculture as growth promoters, antimicrobials, and antioxidants.

Polyphenolic compounds are distinguished by the presence of hydroxyl groups attached to aromatic rings, which confer them significant bioactive properties, most notably antioxidant activity. Moreover, they often exhibit enhanced biochemical activity when complexed with other molecules, compared to their free form [1,2]. Their ability to form physical interactions with a wide range of organic and inorganic compounds not only improves their stability but also enhances their physicochemical properties. As a result, polyphenols are extensively employed as reducing agents, antioxidants, and key components in bioproducts to bolster a myriad of biological effects, including antibacterial, anticarcinogenic, cardioprotective, anti-inflammatory, antifungal, antiviral, and antitoxic activities [3,4].

Polyphenols are also known for their excellent antimicrobial activity against a wide spectrum of bacterial species [3,5,6]. For instance, the in vitro studies carried out by Manandhar et al. (2019) [5] highlighted the potent antibacterial properties of polyphenolic extracts derived from medicinal plants such as *Oxalis corniculata*, *Artemisia vulgaris*, *Cinnamomum tamala*, and *Ageratina adenophora*. These extracts were shown to be highly effective against bacteria, including *E. coli*, *S. aureus*, *S. Typhi*, *P. aeruginosa*, *K. pneumoniae*, and *Rhizopus*. Similarly, Ignat et al. (2013) [6] reported that polyphenols from ethanolic extracts of spruce bark, hawthorn, and grape seed exhibited strong antibacterial activity against both Gram-positive bacteria, like *S. aureus*, and Gram-negative bacteria, such as *P. aeruginosa* and *E. coli*.

Beyond their antimicrobial properties, polyphenolic extracts have also been recognized for their role as plant growth bioregulators and chelating agents [6,7,8,9]. Studies, including those by Ignat et al. (2013) [6] and Tanase et al. (2010) [10], have demonstrated that these compounds can be effectively used to regulate plant growth depending on the source and dosage of the natural compounds applied. Moreover, research has shown that cinnamic and gallic acids can enhance the bioaccumulation capacity of Cd(III) ions in plants, thereby preventing the transport of these harmful ions to the upper parts of the plants. These diverse properties make polyphenolic compounds from biomass extracts highly suitable for a wide range of applications across chemistry, biology, phytotherapy, and environmental engineering [11].

Despite their remarkable potential, polyphenolic compounds face significant challenges due to their poor stability when exposed to different factors such as pH, photo/light, temperature, oxygen availability, metal ions, or enzymes. The polyphenols tend to degrade more when they are exposed to sunlight, temperatures higher than 40 °C, oxygen, and high humidity [12]. This instability requires the development of innovative stabilization techniques to extend their shelf-life and broaden their practical applications. To address these challenges, various methods have been developed to enhance the stability of polyphenols. These techniques include the use of nanoparticles, spray-drying, coacervation, cryopreservation, and encapsulation within liposomes or cyclodextrins [13,14,15], all of which aim to maintain the bioactive properties of polyphenols over extended periods. Additionally, gelation has emerged as a notable method for stabilizing these compounds by integrating phytocompounds into macromolecular polymer networks to create hybrid polymeric gels [15,16,17,18]. These polymeric gels exhibit distinctive properties, including insolubility and infusibility, as well as the remarkable ability to undergo reversible swelling and shrinking in response to environmental changes. These characteristics make them particularly valuable in various applications.

Hydrogels and cryogels, in particular, have garnered significant interest for their potential uses in many fields, including controlled drug and protein delivery, tissue engineering, the separation of small ionic species, and bioseparations [19,20,21,22,23,24,25,26,27,28,29,30,31,32]. In recent research, efforts have been focused on immobilizing natural compounds within hydrogels, with the goal of creating biocompatible and non-toxic materials suitable for use in the pharmaceutical, biomedical, and food industries [15,17,33,34,35,36,37,38,39,40]. This process involves the formation of a protective polymeric matrix that safeguards the biologically active ingredients found in natural compounds, such as vitamins and polyphenols [17,36,37,38,39,40,41,42,43]. The immobilization of natural compounds in polymer cryogels is a growing field of research with scarce but promising data [17,20,33,41,44]. For instance, the incorporation of curcumin into chitosan-based cryogels [33,41] or into chitosan/oxidized starch polyelectrolyte complex sponges [40] has demonstrated a substantial improvement in their biological activities. This enhancement is attributed to the cryogels’ ability to stabilize the natural compound, thereby preserving or even amplifying its bioactive properties. Similarly, successfully integrated papain into poly(hydroxyethyl methacrylate)-chitosan cryogels, enabling its effective use in the purification of apple juice, underscoring the potential of these materials in various biotechnological applications [44].

Polysaccharide-based cryogels, particularly those derived from dextran (Dx), are of significant interest due to their inherent biocompatibility and non-toxic nature [44,45,46]. Dx, a naturally occurring, water-soluble polysaccharide, has seen widespread application across diverse fields, including medicine [44,45] and wastewater treatment [47,48,49], reflecting its adaptability and functional versatility.

In this context, this study aims to enhance the stability of SBE and extend its range of applications by incorporating it into porous Dx-based hydrogels using the freeze-thaw technique. The development of new antioxidant and antimicrobial cryogels based on Dx and SBE holds several distinct advantages. Firstly, the incorporation of SBE in Dx-based cryogels can significantly enhance their stability, thereby extending their functional lifespan. Moreover, when combined with SBE, Dx-based cryogels can form a synergistic system that not only delivers enhanced antioxidant and antimicrobial effects but also ensures safety and minimal environmental impact. From a technological perspective, the freeze-thawing technique used to prepare these cryogels is a simple, cost-effective, and environmentally friendly process [18,23,27,30]. This method aligns with the principles of green chemistry, reducing the need for harmful chemicals and minimizing energy consumption, which is crucial in the development of sustainable materials [18,23,27,30]. Furthermore, the porous structure of cryogels offers a large surface area and high mechanical stability, which are advantageous for the immobilization of bioactive compounds [17,27,39,42]. These structural features enable a better diffusion and interaction of the active compounds with the environment, thereby enhancing the overall efficacy of the material [29,39,40,42]. Thus, the preparation of porous Dx-based hydrogels incorporating SBE through a cryogelation process offers a promising approach for the development of novel biomaterials with enhanced physicochemical and biological properties. The novelty of this study lies in the innovative approach of stabilizing polyphenolic extracts from spruce bark, a byproduct of the wood processing industry, by incorporating them into Dx-based cryogels. This process not only enhances the stability, bioactivity, and bioavailability of the polyphenols but also employs an environmentally friendly method for their encapsulation, a cryogelation technique aligned with the principles of green chemistry. The resulting biomaterials offer enhanced antioxidant and antimicrobial properties, presenting new opportunities for applications in biomedical fields. The combination of SBE and Dx-based cryogels creates a synergistic system with improved functional lifespan, reduced environmental impact, and potential uses in various sectors, including pharmaceuticals, food preservation, and environmental protection. This study represents a pioneering effort to valorize plant biomass waste through sustainable methods, making significant contributions to both material science and waste minimization. To achieve this overall objective, the following specific goals have been proposed: (i) preparation and characterization of SBE; (ii) incorporation of SBE into Dx-based cryogels to enhance its stability, thereby protecting it from external degradation and improving its bioactivity and bioavailability; and (iii) evaluation of the potential applications of Dx cryogels loaded with SBE as antibacterial and antioxidant agents.

## 2. Materials and Methods

### 2.1. Materials

Dx from *Leuconostoc* spp. (molecular weight, M_r_~100.000 g/mol) as a powder was acquired from Fluka and used as received. Ethylene glycol diglycidyl ether (EGDGE, 50 wt.%) was used as a cross-linking agent and was purchased from Sigma-Aldrich. The SBE was prepared from spruce bark *Picea abies* raw material obtained from Șaru Dornei region, Romania. Sodium hydroxide (NaOH, ≥97.0%), as pellets, was purchased from Fluka. Acetic acid (CH_3_COOH, glacial, ≥99.8%), methanol (CH_3_OH, ≥99.9%), ethanol (EtOH, ≥99.5%), sodium carbonate (Na_2_CO_3_, powder, ≥99.5%), and sodium chloride (NaCl, crystals, ≥99%) were also acquired from Sigma-Aldrich (Darmstadt, Germany). Folin Ciocalteu’s phenol reagent was obtained from Fluka, while gallic acid standard ((HO)_3_C_6_H_2_CO_2_H, powder) and phosphate-buffered saline (PBS) were purchased from Sigma-Aldrich.

### 2.2. Preparation and Characterization of Picea abies SBE

To obtain SBE, the lignocellulosic biomass was first milled to a particle size range of 0.5 to 2 mm. The extraction of polyphenols was performed using an ultrasound-assisted method. Specifically, an ultrasound probe (Sonics & Materials INC, Danbury, CT, USA) operating at a power of 600 W and a frequency of 20 kHz was employed. The extraction was carried out with a solid-to-liquid ratio of 1:10 (grams of spruce bark per milliliter of solvent) using 70 wt.% ethanol as the solvent at a running of 10 min.

After extraction, the SBE was concentrated using a rotary evaporator (Heidolph Laborota 4003 Control; Heidolph Scientific Products GmbH, Schwabach, Germany) to remove most of the solvent. The remaining solution was then lyophilized (Christ Alpha 1-4 LSC, Osterode am Harz, Germany) to eliminate any residual ethanol. The resulting powder was subsequently used in the preparation of Dx-based cryogels.

The total polyphenolic content (TPC) of the extract was determined by Folin–Ciocalteu assay, and the data were expressed in milligrams of gallic acid equivalents per gram of spruce bark (mg GAE/g) [17]. The TPC of the SBE used in this study was calculated as 42.6 mg GAE/g.

High-performance liquid chromatography (HPLC) was employed to qualitatively characterize the polyphenolic compounds in the SBE. The analysis was carried out using a DIONEX Ultimate 3000 Chromatographic system equipped with a UV-VIS PDA detector, utilizing an RX C18 Zorbax column (4.5 × 250 mm) with a 5 µm particle size. The chromatographic separation was achieved with two mobile phases: (A) 1% acetic acid in ultrapure water (1:99, *v*/*v*) and (B) ultrapure methanol. The flow rate was set at 1.2 mL/min, and a gradient of 10–40% phase B was applied over 40 min at a constant temperature of 25 °C. Before being injected into the separation column, the SBE was filtered using a 0.45 µm pore size filter. This method enabled the identification and quantification of six phenolic compounds, namely catechin, vanillic acid, syringic acid, sinapic acid, ferulic acid, and p-coumaric acid (Figure S1 and Table S1, Supporting Information), using calibration curves of the respective standards.

### 2.3. Preparation of Dx Cryogels Containing Picea abies SBE

Dx-based cryogels incorporating SBE were prepared as monoliths through the cryogelation approach using ethylene glycol diglycidyl ether (EGDGE) as a cross-linker. To determine the optimal reaction conditions, we systematically varied the initial Dx concentration, the cross-linker content, and the amount of SBE. The initial Dx concentration ranged from 5 *w*/*v*% to 20 *w*/*v*%, the cross-linker amount content varied between 0.28 g/mL and 0.56 g/mL (corresponding to a theoretical cross-linking degree of 1.45 wt.% to 5.6 wt.%), and the amount of SBE ranged from 0.7 g to 1.4 g.

Table 1 presents the sample codes and the composition of each Dx-based cryogel prepared in this work. The cross-linking reaction between Dx and EGDGE was performed in a basic medium by adding a specific amount of 5 M NaOH to the reaction mixture. Before introducing SBE, the pH was adjusted to 10, as a basic environment is crucial for Dx cross-linking with EGDGE, as previously reported [50]. The final solutions remained clear, indicating that the phenolic compounds were not converted to sodium salts. Additionally, the stability of SBE was assessed across a pH range of 1.2 to 12 at room temperature in the dark, with no visible changes observed up to pH 12 (Figure S2). Furthermore, the antioxidant activity of SBE (3.33 mg/mL) in solutions with pH values ranging from 1.2 to 10 showed only a slight decrease during the first three days of investigation (Figure S3). However, by the fifth day, a significant decrease in antioxidant activity was observed, indicating the need for encapsulation in protective materials.

As an example of the synthetic procedure, we provide details for the preparation of DxCG8 cryogels. Typically, 5 mL of 20% dextran was mixed with 0.75 mL of EGDGE 50% and 2 mL of 5 M NaOH to ensure a pH of 10 (Table 1). The cross-linker (EGDGE) was gradually added to the Dx solution under magnetic stirring until full homogenization was achieved. Then, 0.7 g SBE, dispersed in 2 mL of distilled water (pH 4.5–5.0), was added drop–by–drop to the Dx-EGDGE reaction mixture. The resulting mixture containing Dx, EGDGE, and SBE was vigorously stirred for 20 min before being transferred into 1 mL syringes, sealed with parafilm, precooled in liquid nitrogen, and stored at −18 °C using a CC1-K6 Huber Cryostat. After 24 h, the syringes containing the reaction mixture were thawed at room temperature for about 1 h and afterward precooled in liquid nitrogen, then stored again in the cryostat at −18 °C. This precooling–freezing–thawing cycle was performed three times. After the 3rd cycle, the Dx-based cryogels were removed from syringes and cut as monoliths of 10 mm height and immersed in 20 wt.% NaCl solution (about 500 mL) to remove the unreacted compounds. Finally, the Dx-based cryogels containing SBE were dried by lyophilization in a Biobase device for 48 h at −60 °C and 10 Pa. A similar procedure was applied to prepare and dry the cross-linked Dx cryogel without SBE. For purification, the chemically cross-linked Dx cryogels without SBE (control samples) were intensively washed with an excess of MilliQ water (about 500 mL). To quantify the concentration of Dx that was leached from the cryogel, the washing solutions of Dx/SBE cryogel samples were analyzed using the phenol–sulfuric acid method [46,51]. Typically, a 2 mL aliquot of each washing solution was transferred into a colorimetric tube, followed by the addition of 50 mL of 80 wt.% phenol. Subsequently, 5 mL of concentrated H_2_SO_4_ was added rapidly. The tubes were allowed to stand for 10 min before being shaken and placed in a water bath at 25 °C for 20 min. The absorbance of the resulting yellow-orange solution was measured at 490 nm using a SPECORD 200 UV–VIS spectrophotometer (Analytik Jena GmbH+Co. KG, Jena, Germany.). The concentration of Dx, which could leave the cryogel (*C_LDx_*%, Table 1), was determined based on a previously recorded calibration curve.

### 2.4. Characterization of Dx Cryogels Containing Picea abies SBE

The gel fraction yield (*GFY*, %) was calculated by Equation (1) [17,42]:

(1) GFY %=WdWm×100

where *W_d_* is the mass (grams) of the cryogel after purification and lyophilization; *W_m_* is the total mass (grams) of compounds used for cryogel preparation.

The porosity of the cryogels (*P*, %) was determined using the liquid displacement method [36,52], representing the percentage of voids (pores) in a solid material in its swollen state. In this procedure, a defined amount of lyophilized cryogel (~0.01 g) was submerged in a known volume of isopropyl alcohol (*V*_1_) for 5 min. Isopropyl alcohol was selected as the displacement liquid because it is a non-solvent for the Dx matrix, meaning it goes only into the network’s pores without interacting with the cryogel’s components. Following immersion, the total volume of the isopropyl alcohol containing the soaked cryogels was recorded as *V*_2_. After the cryogel sample was removed from the liquid, the remaining volume of isopropyl alcohol was measured as *V*_3_. These volume measurements allowed for the calculation of the cryogel’s porosity (Equation (2)) [36]:

(2) $$P\%=\frac{V_{1}-V_{3}}{V_{2}-V_{3}}\times 100$$

The modification in the chemical structure of the DxCG cryogels was analyzed by Fourier-transform infrared spectroscopy (FTIR) with a Vertex 70 Bruker spectrophotometer. Spectra were recorded in the range of 4000 to 400 cm^−1^, and the data were processed with ACD/Spec Viewer 5.04 software.

The morphological structure of DxCG cryogels was observed with a Quanta 200-FEI environmental scanning electron microscope (ESEM) operating at 20 kV in low vacuum mode. To determine the average pore diameter, images obtained from SEM were analyzed using the Image J 1.48v software. For each sample, the mean pore size was calculated by measuring 50 individual pores, and the results were reported as mean values ± standard deviation (SD) [33]. Detailed information about the elemental composition within the cryogel samples was obtained using energy-dispersive X-ray spectroscopy (EDX).

Swelling studies were conducted by immersing the cryogels in MilliQ water, and the mass of the swollen samples was measured gravimetrically over time until equilibrium swelling was achieved. The swelling ratio (*SR*, g/g) was calculated with Equation (3) [33]:

*SR* = *W_t_/W_d_* (3)

where *W_t_* is the mass of swollen cryogels at time *t*, and *W_d_* is the mass of lyophilized cryogels.

The swelling behavior was also studied under conditions simulating body fluids or food media. Typically, 0.01 g of freeze-dried cryogel was immersed in simulated intestinal fluid (SIF) at pH 7.4, simulated gastric fluid (SGF) at pH 1.2, or a 40% ethanol solution. At predetermined time intervals, the swollen cryogel samples were removed, and their mass was measured using an analytical balance until equilibrium in fluid absorption was reached. The *SR*, g/g was determined using Equation (3).

The stability of the Dx-based cryogels was evaluated as follows: a freeze-dried cryogel sample (0.01 g) was accurately weighed and immersed in weighing vials containing 10 mL of aqueous solutions at different pH levels (1.2, 3, 5, 7, 9, and 12) for 24 h at room temperature. After the immersion period, the cryogel samples were removed from the solution and dried in a vacuum oven at 40 °C until constant weight. The weight loss (*%Wt loss*) was calculated as a percentage using Equation (4) [40]:

(4) $$\%\;Wt\;loss=\frac{W_{0}-W_{t}}{W_{0}}\times 100$$

where *W*_0_ is the initial weight of the cryogel (g), and *W_t_* is the weight of the dried cryogel (g) after 72 h of drying in the vacuum oven at 40 °C.

Uniaxial compression measurements were performed on swollen Dx/SBE cryogels at room temperature using a Shimadzu testing machine (Kyoto, Japan). Cryogel samples, as plates with 10 mm thickness, 12 mm width, and 4 mm height, were compressed with a force of 100 N at a crosshead speed of 1 mm/min. The setup of the test and the calculations of the compressive stress (σ), strain (ε), and elastic modulus were carried out in accordance with the procedure previously reported for other porous biomaterials [53,54].

### 2.5. The Release of SBE from the Dx Cryogels

The release of entrapped SBE from the Dx cryogels was investigated in simulated gastrointestinal (PBS and pH 1.2) and food (40% *v*/*v* EtOH solution) [55] media at different temperatures. The experiments were conducted using samples in which the extract was incorporated either directly in the synthesis stage or by adsorption from ethanolic solution on freeze-dried hydrogels. The encapsulation efficiency (*EE*, %) was calculated with Equation (5) [33]:

(5) $$EE,\;\%=\frac{m_{f}}{m_{i}\;}\times 100$$

where *m_f_* is the weight of SBE (mg) remaining in Dx-based cryogels after purification, and *m_i_* is the weight of SBE (mg) initially added.

The loading of SBE in the Dx cryogels after their synthesis was performed by sorption from hydroalcoholic solution (H_2_O:EtOH, 60:40, *v*/*v*) at a concentration of 10 mg/mL up to maximum sorption capacity. The loaded samples were kept at 4 °C until freeze-drying. Throughout these steps, they were protected from light by covering with aluminum foil. The SBE loading in the Dx cryogels was calculated with Equation (6) [33]:

(6) $$SBE\;loading=\frac{weight\;of\;SBE\;loaded}{weight\;of\;loaded\;Dx\;cryogels}\times 100$$

The weight of SBE loaded in the cryogels was given by the difference between the weight of loaded Dx cryogels to that of initial samples.

The hydrogels containing the SBE were immersed in 10 mL of release medium and shaken in a thermostated water bath (at 37 °C) or an ice bath (at 4 °C). After various time periods, 1.2 mL of supernatant was withdrawn, and the TPC was quantified by Folin–Ciocalteu assay [17,56] as follows: (i) the collected samples were mixed with ethanol until a concentration of 40% (*v*/*v*) was obtained (not applicable when the release of performed in food simulant medium); (ii) 1 mL of the obtained solution was added to a mixture comprised of 1 mL Folin–Ciocalteu reagent and 15 mL water and kept in dark for 6 min; (iii) after the addition of 3 mL of a 20% (*w*/*v*) Na_2_CO_3_ solution, the resulting solution was shaken for 2 h in a thermostated bath at 37 °C in the dark [17]. The absorbance of the final solutions was measured at 764 nm against a blank sample using a SPECORD 200 (Analytik Jena) UV–Visible Spectrophotometer. A gallic acid calibration curve (linear range between 0 and 25 mg/L; R^2^ = 0.9986) obtained by the same protocol was used as a reference. The total polyphenols released (%) from the Dx cryogels was calculated with Equation (7) [39]:

(7) $$Total\;polyphenols\;released=\frac{10\times C_{n}+1.2\times \sum C_{n-1}}{m}\times 100$$

where *C_n_* and *C_n−_*_1_ represent the concentrations of total polyphenols, expressed as mg _GAE_/L, in the releasing medium after *n* and *n* − 1 withdrawing steps; *m* is the amount of polyphenols contained by the tested samples.

### 2.6. Antimicrobial Assay

#### 2.6.1. Bacterial Strains

The bacterial strains used in this study included Gram-positive *Listeria monocytogenes* (ATCC 7644) and Gram-negative *Escherichia coli* (ATCC 25922) and *Salmonella typhimurium* (ATCC 14028), all of which are reference strains.

#### 2.6.2. Determination of Viable Bacterial Cell Numbers by Plate Count (Colony-Forming Units/mL or CFUs/mL)

The antimicrobial assay was conducted following the guidelines outlined in ISO 7218:2007 [57], as previously described [40,58]. To determine the number of viable bacterial cells, serial dilutions of bacterial suspensions were prepared in peptonate physiological serum, adjusted to a turbidity equivalent to 1° on the McFarland scale. This process yielded a final bacterial concentration of 3000 CFUs/mL. The tested cryogel samples were placed in sterile Petri dishes, followed by inoculation with 1 mL of each bacterial dilution. The samples were then incubated at room temperature (25 °C) for 24 h. As a control, sterilized filter paper pieces (1 cm^2^) were also tested under the same conditions. After 24 h of incubation, 100 µL of the inoculum from each sample was extracted and spread onto the surface of specific agar media: XLD agar for Salmonella, ALOA agar for Listeria, and VRBG agar for Escherichia coli. The inoculated plates were then incubated at 37 °C for an additional 24 h. Following incubation, the number of bacterial colonies formed on each plate was recorded, providing a measure of the antimicrobial activity of the cryogel samples (ISO 7218:2007).

### 2.7. Antioxidant Activity Evaluation

To evaluate the antioxidant potential of the Dx-based cryogels, the 2,2-diphenyl-1-picrylhydrazyl (DPPH) radical scavenging assay was employed, following a modified protocol previously described [36,59]. In this procedure, 100 mg of each cryogel sample was added to 3 mL of 40% ethanol and stirred continuously for 30 h to ensure thorough interaction. After this incubation period, the mixture was subjected to ultracentrifugation for 15 min, and the resulting supernatant was collected. For the assay, 500 µL of the cryogel supernatant was mixed with 2500 µL of methanolic DPPH solution (0.06 mM). The mixture was then incubated in the dark for 2 h at 25 °C. The absorbance of the solution was measured at 517 nm to determine the extent of DPPH radical scavenging. For comparison, the antioxidant activity of the SBE was also tested. Three dilutions of the extract were prepared in 70% EtOH, with final concentrations in the cuvette of 0.83 mg/mL, 1.66 mg/mL, and 3.33 mg/mL. Each 500 µL aliquot of these dilutions was mixed with 2500 µL of the DPPH solution (0.06 mM) and incubated for 30 min. The same protocol was followed to evaluate the antioxidant activity of SBE (3.33 mg/mL) in solutions with pH values ranging from 1.2 to 10 over 5 days. The absorbance was then measured at 517 nm. The DPPH radical scavenging activity (%) was calculated by Equation (8):

DPPH radical inhibition (%) = [(A_C_−A_S_)/A_C_] × 100 (8)

where A_C_ represents the absorbance of the control sample (a mixture of 500 µL of 40% EtOH and 2500 µL of DPPH solution), and A_S_ is the absorbance of tested cryogel sample.

The assay was performed in triplicate, and the results were expressed as the mean ± SD. Additionally, the IC_50_ value (mg/mL), which indicates the concentration of extract required to inhibit 50% of the free-radical activity, was determined by linear interpolation between the values that were above and below 50% activity.

## 3. Results and Discussions

### 3.1. Preparation of Dx/SBE Cross-Linked Cryogels

**Figure 1:**
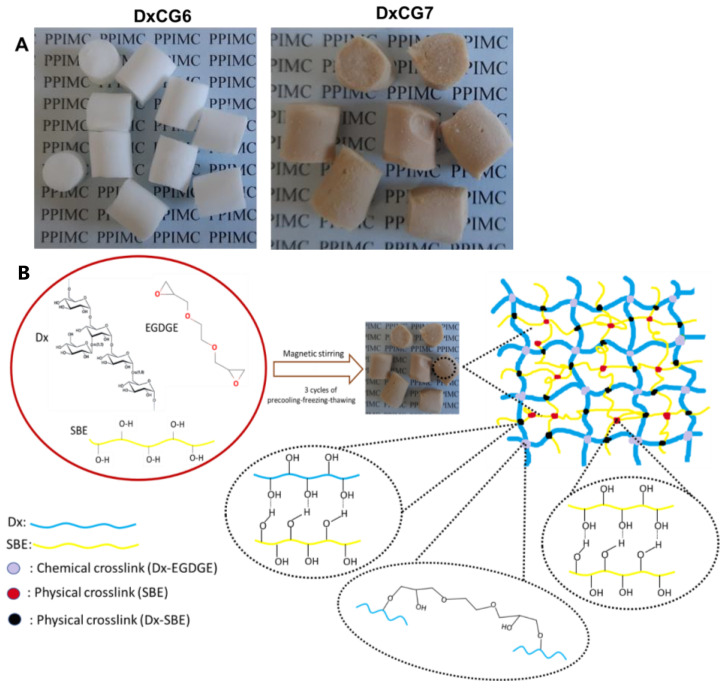
(**A**) Optical images with Dx-based cryogels prepared without SBE (sample DxCG6) and with SBE (sample DxCG7); (**B**) schematic representation of the Dx cross-linking with EGDGE and the interactions through hydrogen bonding between SBE and Dx matrix.

Dual cross-linked cryogels composed of Dx and SBE were successfully developed as monoliths (Figure 1A) using EGDGE as a cross-linker and a precooling freeze-thawing technique simultaneously. EGDGE was chosen as a cross-linker due to several key characteristics: (i) contains epoxide groups at both ends (Figure 1B), which can react with nucleophilic groups from Dx, such as hydroxyl groups, thereby forming stable covalent bonds; (ii) is more water-soluble than many other bis-epoxy compounds, which facilitates better interaction with polysaccharides and proteins in aqueous systems [60,61,62,63,64]; (iii) unlike glutaraldehyde, a commonly used cross-linker that is known for its cytotoxicity [61,65], EGDGE is considered less toxic; (iv) is less expensive and more readily available in bulk compared to many other cross-linking agents, which makes it a practical option for large-scale applications [60]; (v) forms strong and stable networks when used for cross-linking, enhancing the mechanical properties and life-time of the materials; (vi) due to its ability to cross-link a variety of organic macromolecules, including proteins and polysaccharides [60,61,62,63,64], EGDGE has been successfully applied in chromatography, enzymes immobilization, and in the preparation of membranes for biomedical applications.

Dx was selected for its high suitability in biomaterial engineering, attributed to its abundant hydroxyl groups that facilitate the formation of hydrogen bonds with other hydroxyl-rich molecules. This characteristic enables the effective interaction between Dx and the phenolic compounds present in SBE, including catechin, vanillic acid, syringic acid, sinapic acid, ferulic acid, and p-coumaric acid, which were identified and quantified via HPLC (Figure S1, Table S1, Supporting Information). Beyond the physical cross-linking achieved through hydrogen bonding, the chemical cross-linking of Dx with EGDGE was employed to further improve the stability of the incorporated bioactive compounds by creating a more compact three-dimensional (3D) network. This dual cross-linking mechanism, which includes the chemical bonding of Dx and the stabilization of SBE within the Dx matrix through hydrogen bonding, is schematically depicted in Figure 1B. This strategy not only reinforces the cryogel matrix but also ensures the stable incorporation of SBE’s bioactive compounds, thereby enhancing the material’s overall functionality.

The cryogelation approach used to prepare hydrogels relies on the associative interactions between polymer chains from different polymers, as previously reported [36,66,67]. While various types of interactions can lead to polymer association, hydrogen bonding is the predominant mechanism in polysaccharides [36]. This is particularly relevant for Dx, which promotes such interactions due to the abundance of hydroxyl groups present on its backbone. When Dx solutions were frozen in the presence of a chemical cross-linker, such as EGDGE, the liquid water content was significantly reduced to ice forms. This phase transition forces the Dx chains into an ordered, stiff conformation, which, in turn, enhances intermolecular hydrogen bonding, besides the formation of chemical cross-links introduced by EGDGE. Moreover, following the addition of the cross-linker, the resulting Dx solution becomes highly viscous, resembling an intermediate state between a weak gel and a viscous liquid. Then, in the Dx- EGDGE viscous solution, the polyphenolic extract solution is added, and the whole system is frozen at −18 °C for 24 h. During this freezing process, ice crystals form, disrupting the hydrogen bonds between the Dx chains and free water molecules while simultaneously allowing water molecules to interact exclusively with one another. Concurrently, the freezing conditions induce a reorganization of hydrogen bonds between Dx and the polyphenolic extract molecules. It is important to note that some hydroxyl groups on Dx become chemically cross-linked by EGDGE during this time (Figure 1), contributing to the overall stability of the gel. The synthesis of the Dx–polyphenolic extract hydrogel involved a sequential precooling, freezing, and thawing process carried out over three cycles. By applying this protocol, the gel exhibited sufficient cohesion to maintain its structural integrity, which indicates that the density of hydrogen bonds between Dx and the polyphenolic extract was high enough to produce a stable cryogel at room temperature.

To identify the optimal reaction conditions to design stable gels, we systematically modified the initial Dx concentration, the amount of SBE, and the cross-linker content. The results indicate that a Dx concentration of 20% leads to a higher *GFY* (Table 1). For instance, DxCG9 prepared with an initial Dx concentration of 20 wt.% achieved a *GFY* value of 87.78% compared to DxCG3 (10 wt.% Dx) and DxCG1 (5 wt.% Dx), which yielded *GFY* values of 80.66% and 83.00%, respectively. To further validate our results, the samples were purified after synthesis, and the washing solutions were analyzed to determine the concentrations of Dx (*C_LDx_*%, Table 1) and the SBE that may have leached from the Dx-based cryogels. This step was crucial for assessing both the effectiveness of cryogel formation and the encapsulation efficiency (*EE*%, Table 1) of the polyphenolic compounds. A higher Dx concentration accelerates the sol–gel transition to a 3D structure, which allows SBE to be more efficiently entrapped between polymer chains, resulting in *EE* values of 33.14% for DxCG7, 29.21% for DxCG8, and 36.51% for DxCG12 (Table 1). Additionally, the cryogel prepared with 0.7 g of SBE (DxCG7) exhibited a higher *GFY* than those with higher SBE content (DxCG8). This result aligns with the incomplete encapsulation of polyphenols and the levels of Dx leached from the 3D network during purification (*C_LDx_* values of 9.01%, 11.08%, and 5.37% for DxCG8, DxCG10, and DxCG12, respectively; see Table 1). These findings are also supported by previous research on cryogel films based on xanthan gum and poly(vinyl alcohol) cryogels loaded with red grape pomace [67].

In terms of cross-linker content, a lower concentration of EGDGE resulted in a reduced *GFY* (Table 1), likely due to insufficient cross-linking to stabilize the Dx molecules. When considering Dx cryogels without SBE, prepared with an initial Dx concentration of 20 wt.%, increasing the cross-linker amount from 0.28 g/mL to 0.42 g/mL raised the *GFY* from 87.78% (DxCG9) to 96.4% (DxCG6). However, further increasing the cross-linker amount to 0.56 g/mL slightly decreased the *GFY* to 91.1% (DxCG11). On the other hand, higher EGDGE concentrations positively impacted SBE incorporation into the Dx structure, as demonstrated by the *GFY* increase from 57.34% (DxCG10) to 62.19% (DxCG8) and 69.62% (DxCG12) (Table 1), possibly due to a denser network that facilitated more efficient SBE integration.

### 3.2. Structural and Elemental Characterization of Dx/SBE Cross-Linked Cryogels

**Figure 2:**
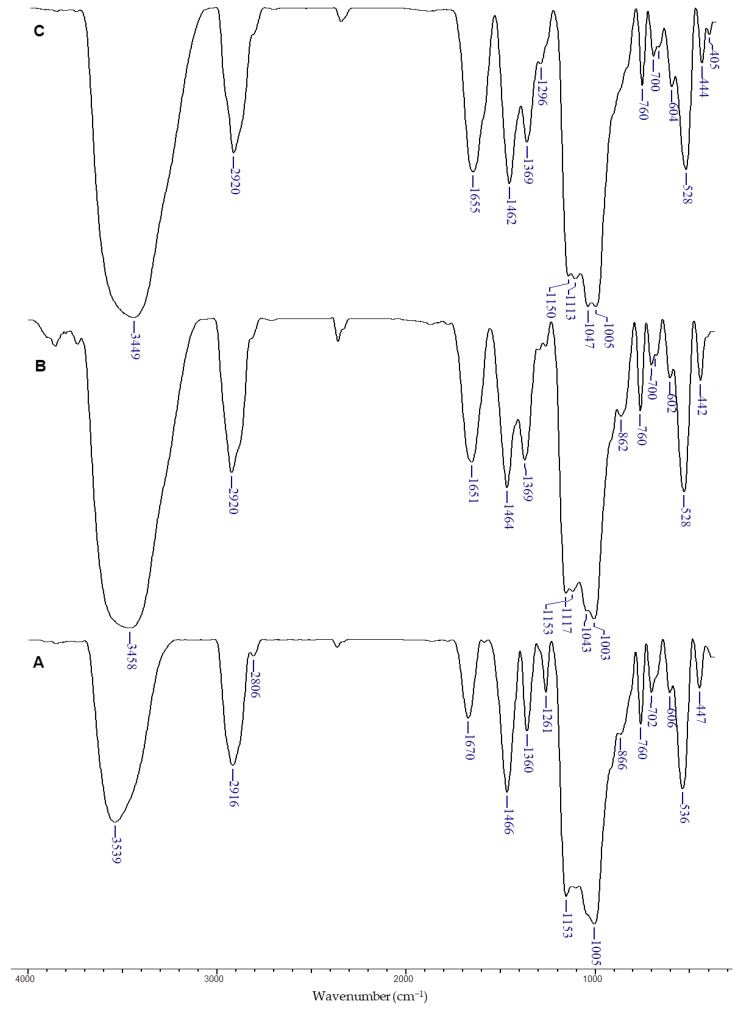
FTIR spectra of DxCG6 (**A**), DxCG7 (**B**), and DxCG8 (**C**).

FTIR spectroscopy was employed to characterize both the Dx-based cryogels and those loaded with SBE. The FTIR analysis, as shown in Figure 2, reveals the complex compositional structure of these cryogels, highlighting the presence of aromatic groups from polyphenolic compounds and polymer chains associated with Dx.

The main characteristic bands of Dx were identified in the FTIR spectrum of the DxCG6 cryogel (Figure 2A), with key absorption peaks observed at 3539 cm^−1^ (O–H stretching vibrations), 2916 cm^−1^ and 1466 cm^−1^ (-CH_2_ group vibrations), 1360 cm^−1^ (-CH bending vibrations), and 1261 cm^−1^ (primary O–H in-plane bending), along with a peak at 1005 cm^−1^ (C-O stretching vibrations in the anhydroglucose (AGU) ring [35,47]. It should be pointed out that the absorption peak corresponding to the C-O-C bridge in AGU units, initially at 988 cm^−1^ in Dx powder (Figure S4, Supporting Information), was red-shifted to 1005 cm^−1^ in the DxCG6 cryogel (Figure 2A), indicating successful cross-linking with EGDGE (Figure 1B). Additionally, a distinct peak at 1153 cm^−1^ supports the formation of supplementary ether (C–O–C) and hydroxyl (-OH) groups [50,68].

In the FTIR spectrum of SBE (Figure S5, Supporting Information), characteristic absorption bands were observed, corresponding to hydroxyl groups (3200–3600 cm^−1^), C-H stretching vibrations in alkyl chains (2850–2950 cm^−1^), carbonyl groups (1630–1750 cm^−1^), C=C stretching in aromatic rings (1500–1600 cm^−1^), and C-O stretching vibrations in alcohols, ethers, and esters (1000–1300 cm^−1^), as well as out-of-plane C-H bending in aromatic rings (700–900 cm^−1^) [69,70,71].

When SBE was incorporated into the DxCG cryogels, important shifts and changes in the FTIR spectra were observed. For instance, the O–H stretching vibration band was blue-shifted from 3539 cm^−1^ in Dx to 3458 cm^−1^ (Figure 2B, DxCG7) and 3449 cm^−1^ (Figure 2C, DxCG8) in the Dx/SBE cryogels. Additionally, the primary O–H in-plane bending band at 1261 cm^−1^ was significantly diminished in the FTIR spectrum of the Dx/SBE cryogels, indicating strong interactions between the hydroxyl groups of Dx and the phenolic compounds in SBE (Figure 2). These spectral changes were consistent across cryogels prepared with varying Dx concentrations (Figure S6, Supporting Information) and cross-linker amounts (Figure S7, Supporting Information), confirming the effective incorporation of SBE within the Dx matrix.

**Figure 3:**
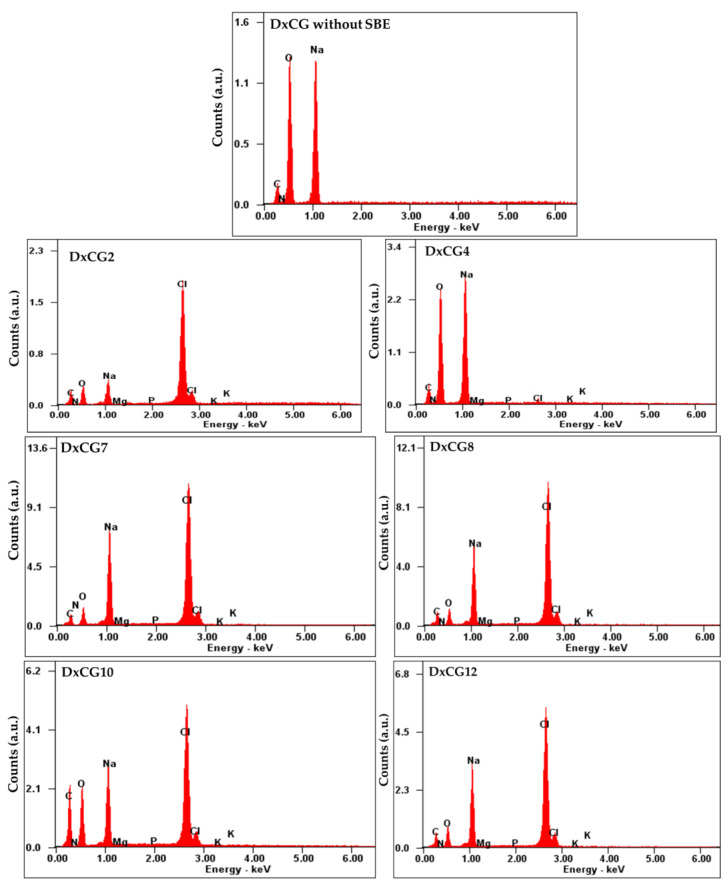
EDX profiles for Dx-based cryogels prepared without or with BSE.

EDX analysis is an analytical technique for determining the elemental composition of various materials, such as metals, ceramics, biological tissues, and complex compounds [72]. EDX is useful for mapping the spatial distribution of elements and detecting trace elements or contaminants. In the context of composite materials, such as biomaterials or hydrogels, EDX can help confirm the presence of specific compounds, additives, or fillers, as in the case of SBE within Dx cryogels. Thus, EDX measurements were performed on the surfaces of Dx-based cryogels, both with and without SBE (Figure 3 and Tables S2, Supporting Information).

The surface mapping showed that SBE was successfully trapped in the Dx matrix. As a result, the Dx/SBE cryogels consisted of carbon (C), oxygen (O), magnesium (Mg), phosphorus (P), and potassium (K) (Figure 3, Table S2, Supporting Information). Mg, P, and K elements are coming from SBE. EDX profiles also detected sodium (Na), which was expected to occur due to the cross-linking reaction that takes place in a basic environment. In addition, chlorine (Cl) was observed in various weight percentages (Table S2, Supporting Information), likely due to extensive washing of the Dx/SBE cryogels with a NaCl solution to remove unreacted components.

### 3.3. Morphology, Pore Sizes, and Porosity of Dx/SBE Cross-Linked Cryogels

**Figure 4:**
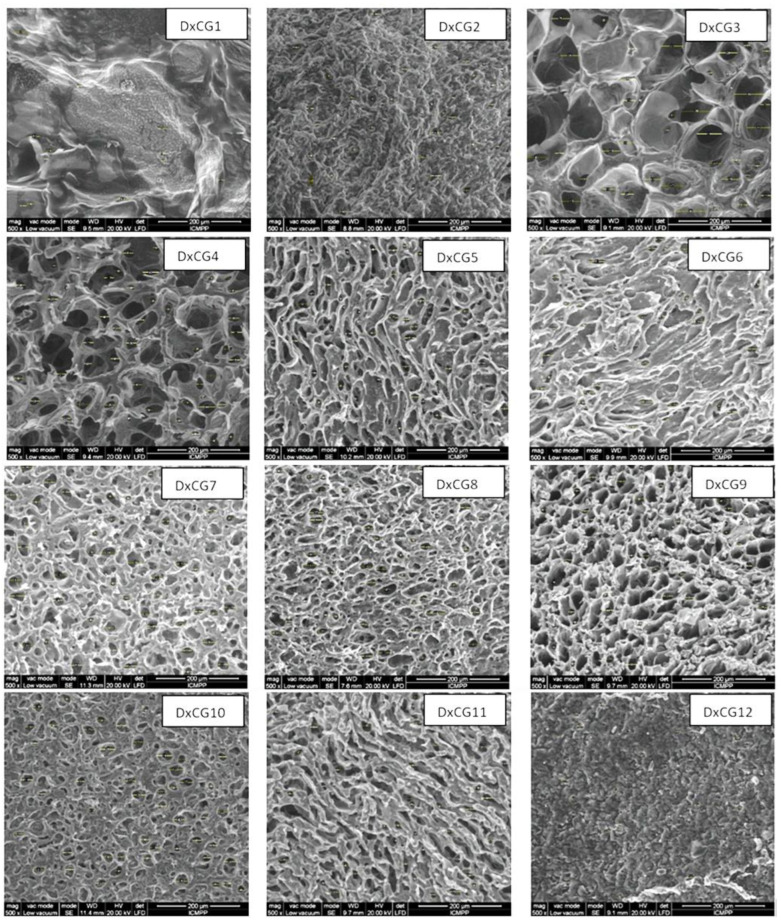
SEM micrographs of Dx cryogels prepared without and with SBE.

**Figure 5:**
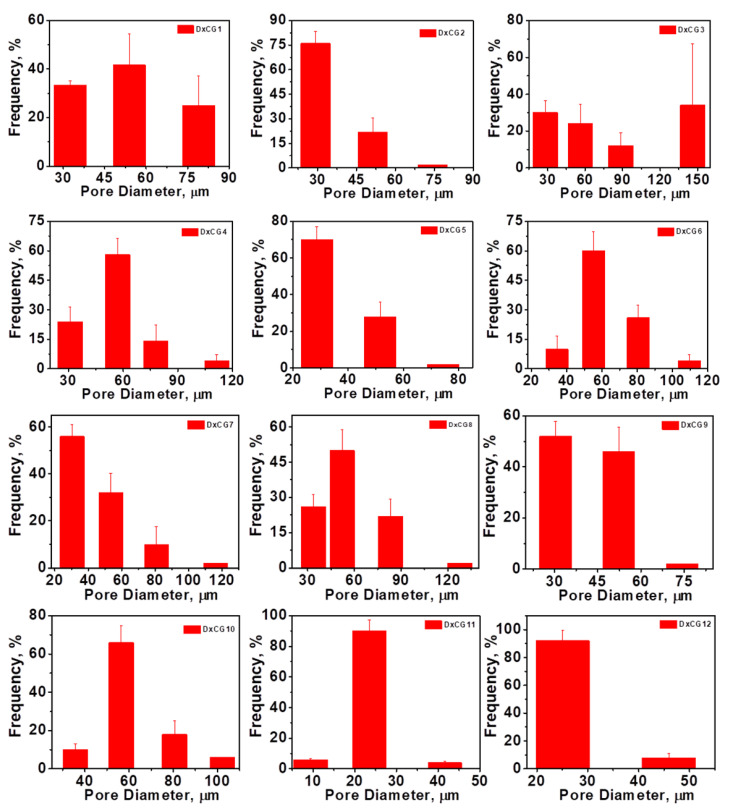
Pore size distribution for Dx-based cryogels.

The properties of Dx/SBE cross-linked cryogels are tightly connected to their morphology, and in this regard, the internal structure of these biomaterials was analyzed by SEM. The SEM images showed that the internal structure of Dx/SBE cryogels is markedly different from that of Dx cryogels without SBE (Figure 4). The incorporation of SBE within the Dx matrix resulted in denser structures with thicker pore walls, indicating a higher degree of cross-linking in Dx/SBE cryogels. The pore sizes within the cryogels were found to decrease with increasing concentrations of Dx, EGDGE (cross-linker), and SBE (Figure 5). This trend suggests that higher concentrations promote the formation of more cross-links, leading to a more compact structure.

The SEM analysis revealed a heterogeneous porous morphology across all Dx-based cryogels, with pore sizes varying as a function of the initial Dx concentration and cross-linker content. For instance, when the Dx concentration was increased from 5 wt.% (sample DxCG1, Figure 4) to 20 wt.% (sample DxCG9, Figure 4), the cryogels exhibited a honeycomb structure with a uniform distribution of pores.

The incorporation of SBE further influenced the pore structure, resulting in smaller and more uniformly distributed pores, particularly when higher amounts of SBE were used (as shown for samples DxCG8 and DxCG10, Figure 4). Similarly, increasing the cross-linker content led to a decrease in pore size and a smoother overall structure, as evidenced by the samples with higher EGDGE content (samples DxCG8, DxCG10, DxCG12, Figure 4 and Figure 5). In cryogels prepared with lower concentrations of Dx or cross-linker, a wide distribution of pore sizes was observed. However, at higher Dx concentrations, there was a notable increase in the frequency of smaller pores, particularly in the 20–60 µm range (sample DxCG9, Figure 5).

Regarding porosity, it was observed that the porosity of the cryogels decreased as the Dx concentration increased, with a significant drop from 78.9% in cryogels with 5 wt.% Dx (DxCG1, Table 1) to 58.74% in those with 20 wt.% Dx (DxCG9, Table 1). However, the amount of cross-linker did not significantly impact porosity. Interestingly, cryogels with higher SBE content exhibited the highest porosity values, around 96% (DxCG10, Table 1), due to the presence of numerous small, well-distributed pores on the cryogel surface. In summary, the morphology and pore structure of the Dx/SBE cryogels are highly dependent on the concentrations of Dx, EGDGE, and SBE, with higher concentrations leading to denser structures with smaller pores. The porosity also varies significantly with Dx and SBE content, highlighting the ability to tailor these properties through careful adjustment of the preparation parameters.

### 3.4. Swelling Behavior of Dx/SBE Cross-Linked Cryogels

**Figure 6:**
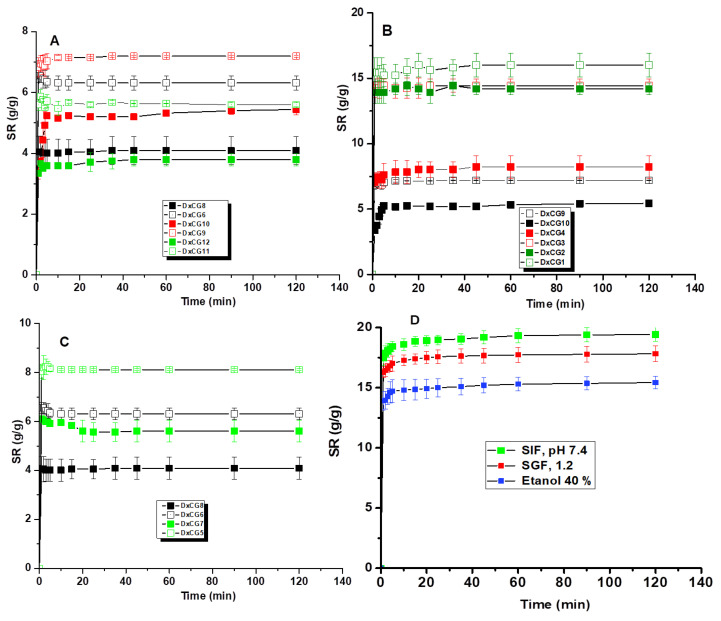
Swelling kinetics of Dx-based cryogels differ by EDGDE content (**A**); initial Dx concentration (**B**) and SBE amount (**C**). The swelling kinetics of Dx/SBE cryogels are presented in comparison with those of Dx cryogels without SBE prepared in similar conditions. (**D**) Swelling kinetics of DxCG12 under conditions simulating body fluids (SIF at pH 7.4 and SGF at pH 1.2) or food media (ethanol 40%). All data are represented as mean value ± SD.

The swelling behavior of cryogels reveals their capacity to absorb and retain water or other fluids, which is also indicative of their long-term stability and durability [42]. Excessive swelling can compromise the cryogel’s structural integrity, leading to breakdown, while controlled swelling suggests a more durable material capable of maintaining its function over time [36]. Therefore, the swelling kinetics of Dx-based cryogels were assessed in MilliQ water (Figure 6).

The swelling ratio (*SR*) for all Dx-based cryogels increased over time until reaching equilibrium (Figure 6). As expected, increasing the amount of EGDGE cross-linker resulted in lower *SR* values (Figure 6A). For instance, cryogels prepared with 0.28 g/mL (sample DxCG9), 0.42 g/mL (sample DxCG6), and 0.56 g/mL EGDGE (sample DxCG11) exhibited *SR* values of 7.19 g/g, 6.31 g/g, and 5.6 g/g, respectively. In Dx cryogels containing SBE and the same amount of cross-linker, the *SR* values were slightly lower at 5.44 g/g (DxCG10), 4.09 g/g (DxCG8), and 3.79 g/g (DxCG12).

An increase in the initial Dx concentration from 5 wt.% to 20 wt.% at a constant EGDGE amount of 0.28 g/mL also led to a reduction in the cryogels’ water content. Consequently, *SR* values dropped from 16.02 g/g (sample DxCG1, Figure 6B) and 14.19 g/g (sample DxCG2, Figure 6B) to 7.19 g/g (sample DxCG9, Figure 6B) and 5.44 g/g (sample DxCG10, Figure 6B).

The amount of SBE incorporated into the Dx-based cryogels further influenced their water retention capacity, with higher SBE content leading to lower *SR* values (Figure 6C). Specifically, *SR* values decreased from 8.12 g/g (DxCG5, Figure 6C) and 6.31 g/g (DxCG6, Figure 6C) to 5.61 g/g (DxCG7, Figure 6C) and 4.09 g/g (DxCG8, Figure 6C). Given the hydrophobic nature of the natural compounds in SBE, the reduced swelling capacity of DxCG can be attributed to a diminished hydrophilic character of the cryogels incorporating higher amounts of SBE. Similar results were also previously reported for other porous polysaccharide materials incorporating polyphenolic compounds [17,67].

Exploring the behavior of materials in simulated gastrointestinal fluids or food media is essential for potential applications in sustained drug delivery or food packaging. Therefore, the swelling kinetics of DxCG12 was investigated under conditions simulating body fluids (SIF at pH 7.4 and SGF at pH 1.2) or food media (40% ethanol) (Figure 6D). Swelling equilibrium was reached in less than 5 min, comparable to that of other macroporous polysaccharide-based hydrogels [15,39,46]. The DxCG12 cryogel exhibited a higher swelling ratio at pH 7.4 than at pH 1.2 or in ethanol 40%, as the -COOH groups from polyphenolic acids are converted to -COONa, enhancing the hydrophilic nature of the matrix. The swelling study of Dx/SBE cross-linked cryogels demonstrates a clear relationship between the composition of the cryogels and their ability to absorb water. Increasing the concentration of the cross-linker EGDGE results in a decrease in the *SR*, indicating that a denser network with more cross-links limits water absorption. Similarly, a higher initial concentration of Dx and greater incorporation of SBE also lead to reduced swelling, suggesting that these factors contribute to a more compact structure, as was also observed by SEM analysis (Section 3.3, Figure 4).

### 3.5. Mechanical Properties of Dx/SBE Cross-Linked Cryogels

Uniaxial compression tests were conducted on swollen DxCG cryogel samples to evaluate their mechanical properties. The stress–strain profiles for DxCG6, DxCG8, DxCG11, and DxCG12 cryogels are displayed in Figure S8. The compressive elastic modulus was determined from the slope of the linear region of the stress–strain curves, following a standard procedure used for other polysaccharide-based cryogels [17,33,39,40,53,54]. The values for compressive nominal stress (kPa), strain (%), and elastic moduli are listed in Table 2.

As depicted in Figure S8, all DxCG samples displayed typical compressive stress–strain behavior for porous materials prepared via freeze-thawing. The DxCG cryogels withstood compressive strain of over 84% without fracturing, indicating the formation of a relatively dense cross-linked network and the complete expulsion of water from macropores during compression. Similar mechanical behavior has been reported for hybrid cryogels comprising functionalized chitosan and St. John’s Wort extract [17], as well as chitosan/dextrin cryogels cross-linked with *Thymus vulgaris* essential oil [36]. An increase in cross-linker ratio led to enhanced compressive strength, rising from 679.72 kPa for DxCG6 to 1003.93 kPa for DxCG12. However, the incorporation of SBE resulted in a stiffer matrix, which decreased both the compressive strength and elastic modulus values. This stiffening effect suggests that SBE influences the mechanical characteristics of the cryogels, with a significant influence on matrix flexibility.

### 3.6. Stabilization of SBE within Dx-Based Hydrogels

**Figure 7:**
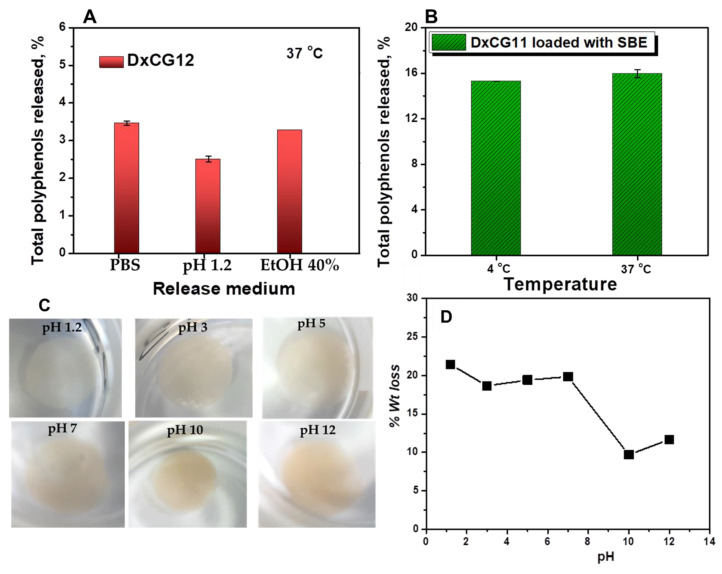
The total spruce bark polyphenols released from Dx cryogels in 24 h as a function of encapsulation strategy: (**A**) in different release media at 37 °C from DxCG12 cryogel in which the SBE was incorporated directly from the preparation process and (**B**) in PBS at different temperatures from DxCG11 cryogel in which the SBE was loaded by post-synthesis adsorption. (**C**) Optical images of the DxCG12 cryogel sample after 24 h of immersion in aqueous solutions at various pH levels. (**D**) The weight loss of the cross-linked DxCG12 cryogel as a function of pH.

The ability of Dx matrices to stabilize polyphenols in SBE was investigated by evaluating the total polyphenols released from Dx cryogels under simulated gastrointestinal conditions (PBS or pH 1.2 aqueous solutions) and food-related conditions (40% ethanol solution) at 37 °C in the dark. These conditions help preserve the stability of the polyphenols, as well as their initial encapsulation within the hydrogels. These aqueous environments were shown to not affect the appearance (Figure S2) and the DPPH radical scavenging activity of SBE, at least in the first three days of investigations (Figure S3). Furthermore, the primary polyphenols in SBE, such as catechins and polyphenolic acids (e.g., sinapic and ferulic acids), were found to be more stable under these conditions. As reported in the literature, these compounds are more susceptible to degradation when exposed to light [73]. The release experiments were designed to also evaluate the role of the encapsulation strategy on the extract stabilization efficiency by using samples in which the extract was introduced either directly in the precursor reaction mixture or by adsorption from ethanolic solution on freeze-dried cryogels. Figure 7 presents the total polyphenols release determined by the Folin–Ciocalteu assay from two different Dx cryogels under various release conditions. Figure 7A shows release data for a Dx cryogel (DxCG12) in which the SBE was loaded directly from synthesis (with an *EE* of 36.51%, Table 1), whereas Figure 7B shows the release data from a Dx cryogel (DxCG11) in which SBE was loaded after the gel was synthesized (with an SBE loading of 73.82%). Although we tested different release conditions, for comparison purposes, we also investigated the total polyphenols released from both cryogels in PBS at 37 °C (Figure 7).

As anticipated, the release of SBE from the Dx cryogels in which it was incorporated directly from the synthesis stage was lower (up to ~3–4%) (Figure 7A) than those in which the extract was adsorbed on the freeze-dried gels (~15–16%) (Figure 7B). This shows that the addition of the extract directly in the precursor reaction mixture, and its subjection to cryostructuration enables a more efficient inclusion of the polyphenols in the pore walls of the cryogels. The release medium (PBS, pH 1.2, or 40% EtOH) (Figure 7A) or the temperature (Figure 7B) had a minor influence on the total polyphenols released from the tested cryogels. Overall, it can be summarized that both strategies enabled the stable encapsulation of SBE in the Dx cryogels, indicating the establishment of strong interactions between the hydroxyl groups of the constituent polyphenols and the functional groups of Dx. Release rates of up to ~11.5% were recently reported by Borges-Vilches et al. for *Pinus radiata* bark extract from freeze-dried chitosan scaffolds, which was mainly attributed to the formation of a high number of hydrogen bonds between the hydroxyl groups of the constituent polyphenols and the functional groups of the polysaccharide [74]. In addition, other types of physical interactions, such as π-π interactions [15] could also be involved in the stabilization of the polyphenols in the SBE in the Dx cryogels. As shown in the optical images in Figure 7C, the DxCG12 cryogel remains stable after 24 h of immersion in aqueous solutions across a range of pH values, without any signs of disintegration, regardless of the solution’s pH. Further evaluation of the cryogel’s stability was performed by drying it to a constant weight. Figure 7D highlights that DxCG12 maintains high stability in an alkaline environment, with only 9–12% weight loss. In contrast, at a highly acidic pH of 1.2, the cryogel exhibited a slightly higher weight loss of around 21%.

### 3.7. Antimicrobial Activity

The antibacterial activity of the cryogel samples and *Picea abies* SBE was assessed against Gram-positive and Gram-negative strains by determining viable bacterial cell numbers using the plate count method. The results are presented in Table 3.

The cryogel samples containing SBE and the extract itself showed remarkable activity (100% inhibition of bacterial growth) on both Gram-positive and Gram-negative bacterial strains. They were more effective as an inhibitor of bacterial growth colonies compared to the Dx-based cryogels without SBE. Antimicrobial activity of different SBE (aqueous, ethanolic) has been reported [75]. *Escherichia coli* bacterium is one of the most susceptible strains to the action of spruce bark aqueous extract [76]. The antimicrobial effects are significantly linked to the chemical composition of the extracts. In our case, the extract of *Picea abies* bark contains phenolic compounds such as phenolic acids and catechins. They are known to exhibit potent antimicrobial properties, acting by various mechanisms, namely permeabilization of the cytoplasmatic membrane, bacterial enzyme and nucleic acids inhibition, cytoplasm acidification, ions leakage, and the alteration of physicochemical surface properties of bacterial cells [77,78]. Different studies reported an important antimicrobial activity of syringic [79,80], ferulic [77], sinapic [81], and vanillic acids [79], the main phenolic acids identified in our *Picea abies* SBE. Among hydroxybenzoic acid derivatives, syringic acid inhibited the growth of *Escherichia coli*, *Salmonella typhimurium*, and *Staphylococcus aureus* bacteria, including multidrug-resistant strains. It affects cell membrane permeability, inducing ion leakage and proton influx [80]. Also, ferulic acid, a derivative of hydroxycinnamic acid, exhibits activity against both Gram-positive and Gram-negative bacteria, including *Listeria* and *Escherichia coli* pathogens. It alters bacterial membrane properties (charge and intra- and extracellular permeability), causing a pore formation in the cell membranes and the leakage of vital intracellular constituents [77]. Due to their propenoid side chain, hydroxycinnamic acids are more lipophilic than the corresponding hydroxybenzoic acids, and they can easily diffuse through bacterial membranes, allowing good activity [77,79,82]. Other compounds of SBE, such as catechins, are also recognized for their high antibacterial activity, and they have been more effective on Gram-positive bacteria than Gram-negative strains. These phenolic compounds cause membrane damage; they intercalate into the lipid bilayer and lead to bacterial membrane disruption [78]. It is noteworthy that the cryogel samples containing SBE are as effective as the extract on the investigated bacterial strains known as hazardous foodborne pathogens [83]. Thus, the limitations of the extract use, such as those related to the low stability and short shelf-life, can be controlled. The inclusion of SBE in such formulations prevents its degradation and the risk of loss of antimicrobial efficacy.

### 3.8. Antioxidant Activity

The free radical scavenging properties of Dx-based cryogels containing *Picea abies* SBE were evaluated using the DPPH assay. The antioxidant activity of the extract itself was also determined using the same test. All results are shown in Table 4.

The cryogel sample without SBE (sample DxCG6) did not show antioxidant activity. The Dx-based cryogels containing *Picea abies* SBE showed moderate free radical scavenging properties; the highest antioxidant activity was observed for the sample containing the highest concentration of SBE (sample DxCG8) (Table 4). Bark of *Picea abies* is an important forestry waste material and a source of valuable antioxidant polyphenols as flavonoids (catechins, taxifolin, quercetin), polyphenolic acids, and stilbenes (piceid) [84]. In our study, the total extract of (*Pinus*) *Picea abies* bark presented a good DPPH scavenging capacity (over 70% inhibition of DPPH radical at 3.33 mg/mL). This value is higher compared to the results reported by Nisca et al. (2021) [84] for the hydroalcoholic SBE also attained by ultrasound-assisted extraction (78.17% vs. 52.64%). The plant products, the geographical origin, the age of the plant, the harvesting method, and the type of extract have an important influence on the content and spectrum of antioxidant polyphenols. All these factors and also the methodology of antioxidant assay can explain the differences from other studies.

The determination of antioxidant activity provides a straightforward method to assess the stability of phenolics. Additionally, comparing the UV spectra and strong absorption peaks of compounds in the release media with those of standards can facilitate an indicative assessment of potential degradation. For instance, a difference of less than 10 nm may be attributed to the substitution of hydroxyl groups on the aromatic ring, which leads to a bathochromic shift (λ_max_ value) towards longer wavelengths due to the electron-donating effect of these groups [85]. In our case, the lower activity of the formulations compared to the extract per se may be related to the involvement of some of the chemical groups relevant to the antioxidant activity in hydrogen bonding with hydroxyl groups of Dx during the preparation of cryogels. The presence of -CH=CH-COOH groups in the hydroxycinnamic acids and the carboxyl group in the hydroxybenzoic acids is important for their antioxidant activity. The -CH=CH-COOH groups in hydroxycinnamic acids provide higher H-donating ability and subsequent radical stabilization. The free radical scavenging activities of simple catechins are attributed to the presence of the ortho-dihydroxyl group in the B ring [86].

## 4. Conclusions

Porous hydrogels composed of Dx and SBE, were successfully synthesized using a dual cross-linking method, which included chemical cross-linking with EGDGE and physical stabilization through freeze-thawing. FTIR analysis revealed the interactions between the functional groups of Dx and the phenolic compounds in SBE through hydrogen bonding. SEM images showed denser cryogels with smaller, uniformly distributed pores as Dx, EGDGE, and SBE concentrations increased, indicating enhanced cross-linking and structural stability. Swelling studies indicated controlled water absorption, which decreased as the Dx, EGDGE, and SBE concentrations increased, suggesting a denser network that limits water uptake. Release studies of SBE indicated effective stabilization, with only 3–4% release when SBE was incorporated during synthesis and 15–16% when loaded post-synthesis. Cryogels containing SBE exhibited remarkable antimicrobial activity against both Gram-positive and Gram-negative bacteria, attributed to SBE’s phenolic compounds. Additionally, these cryogels demonstrated strong antioxidant activity, slightly lower than the SBE extract itself, possibly due to some antioxidant groups being involved in cross-linking.

In summary, the dual cross-linked Dx-based cryogels demonstrated effective stabilization and incorporation of SBE, resulting in biomaterials with controlled swelling and outstanding antimicrobial and antioxidant properties, which could have potential applications in several areas, including wound dressings or scaffolds in tissue engineering and coatings for medical devices, implants, or surfaces that require protection against bacterial contamination. The unique antioxidant and antimicrobial properties make these cryogels promising candidates for food packaging materials to extend shelf life and prevent spoilage by inhibiting the growth of bacteria and reducing oxidation.

## Figures and Tables

**Table 1:** The sample codes and the compositions of Dx-based cryogels (DxCG).

Sample Code	Dx, wt.%	SBE, g	H_2_O, mL	^a^ EGDGE, g/mL	^b^*GFY*, %	^c^*EE*, %	^d^*C_LDx_*, %	^e^*P*, %
DxCG1	5	0	2	0.28	83.00	-	11.16	78.9
DxCG2	5	1.4	-	0.28	41.9	6.22	13.86	77.31
DxCG3	10	0	2	0.28	80.66	-	12.47	72.96
DxCG4	10	1.4	-	0.28	60.63	10.85	11.95	83.52
DxCG5	20	0	1	0.42	94.23	-	2.88	48.36
DxCG6	20	0	2	0.42	96.4	-	2.05	56.25
DxCG7	20	0.7	-	0.42	75.97	33.14	8.72	55.01
DxCG8	20	1.4	-	0.42	62.19	29.21	9.01	57.23
DxCG9	20	0	2	0.28	87.78	-	5.63	58.74
DxCG10	20	1.4	-	0.28	57.34	12.74	11.08	96.36
DxCG11	20	0	2	0.56	91.1	-	3.46	54.27
DxCG12	20	1.4	-	0.56	69.62	36.51	5.37	94.24

^a^ EGDGE with a concentration of 50 wt.%; ^b^ *GFY*—gel fraction yield was calculated with Equation (1); ^c^ *EE%*—encapsulation efficiency was calculated with Equation (4); ^d^ *C_LDx_*%—concentration of Dx that was leached from the cryogel during the washing step; ^e^ *P*—porosity was evaluated with Equation (2).

**Table 2:** Compressive nominal stress and elastic moduli of the equilibrium swollen Dx/SBE cross-linked cryogels.

Sample Code	Compressive Nominal Stress, kPa	Strain %	Compressive Elastic Modulus, kPa	R^2^
DxCG6	679.72	90.90	15.40	0.997
DxCG8	404.43	84.58	5.43	0.998
DxCG11	1003.93	97.73	25.22	0.997
DxCG12	383.64	86.61	8.18	0.998

**Table 3:** The inhibition of bacterial growth exhibited by Dx-based cryogels containing hydroalcoholic extract from *Picea abies* bark.

Microorganisms		Inhibition of Bacterial Growth (%)			
		DxCG6	DxCG7	DxCG8	SBE
Gram(+)	*Listeria monocytogenes* ATCC 7644	51	100	100	100
Gram(−)	*Escherichia coli* ATCC 25922	27	100	100	100
Gram(−)	*Salmonella typhymurium* ATCC 14028	67	100	100	100

**Table 4:** DPPH radical scavenging activity of hydroalcoholic extract from *Picea abies* bark and Dx-based cryogels containing *Picea abies* bark extract.

Samples	DPPH Radical Inhibition (%)	IC_50_ (mg/mL)
DxCG6	0	-
DxCG7	30.37 ± 0.11	-
DxCG8	42.19 ± 0.29	-
SBE 3.33 mg/mL	78.17 ± 0.19	1.76 ± 0.05
SBE 1.66 mg/mL	54.12 ± 0.12	
SBE 0.83 mg/mL	17.11 ± 0.11	

## Data Availability

Data are contained within the article and Supplementary Materials.

## Supplementary Materials

The following supporting information can be downloaded at: https://www.mdpi.com/article/10.3390/polym16192834/s1, Figure S1. The chromatographic profile of SBE; Table S1. HPLC analysis of SBE; Figure S2. Optical images of SBE in solutions with pH ranging from 1.2 to 12 show no visible changes in appearance up to pH 12. At pH 12, the SBE solution darkens, indicating the oxidation of polyphenolic compounds present in the SBE; Figure S3. DPPH radical scavenging activity of hydroalcoholic extract from *Picea abies* bark in solutions with pH values ranging from 1.2 to 10 over 5 days; Figure S4. FTIR spectrum of Dx powder; Figure S5. FTIR spectrum of SBE; Figure S6. FTIR spectra of Dx-based cryogels prepared with different Dx initial concentrations; Figure S7. FTIR spectra of Dx-based cryogels prepared with different cross-linker amounts; Table S2. Weight percentages (wt.%) of elements found on the surface of Dx-based cryogels. Values reported are the average of two measurements; Figure S8. Representative stress-strain profiles for the DxCG cryogels without (DxCG6 and DxCG11) and with SBE (DxCG8 and DxCG12) obtained by applying a force of 100 N under a displacement control rate of 1 mm min^−1^.
