# Supplementary material for: Stabilization of Picea abies Spruce Bark Extracts within Ice-Templated Porous Dextran Hydrogels
Source: Polymers (Basel). 2024 Oct 7;16(19):2834. doi: 10.3390/polym16192834 (PMC11478723; doi:10.3390/polym16192834)
Supplement: Supplementary file 1 [file polymers-16-02834-s001.zip › polymers-3195704-supplementary.pdf]

Supporting information

STABILIZATION OF *PICEA ABIES* SPRUCE BARK EXTRACTS WITHIN ICE-TEMPLATED POROUS DEXTRAN HYDROGELS

Roxana Petronela Damaschin [1], Maria Marinela Lazar [2], Claudiu-Augustin Ghiorghita [2], Ana Clara Aprotosoaie[3], Irina Volf [1,*] and Maria Valentina Dinu [2,*]

1 "Cristofor Simionescu" Faculty of Chemical Engineering and Environmental Protection, "Gheorghe Asachi" Technical University of Iasi, Prof. Dimitrie Mangeron Boulevard 73, Iasi 700050, Romania; roxanadamaschin.rd@gmail.com

2 "Petru Poni" Institute of Macromolecular Chemistry, Grigore Ghica Voda Alley 41A, Iasi 700487, Romania; maria.lazar@icmpp.ro (M.M.L.); claudiu.ghiorghita@icmpp.ro (C.A.G.)

3 "Grigore T. Popa" University of Medicine and Pharmacy, Faculty of Pharmacy, Universitatii Street 16, Iasi 700115, Romania; claraaprotosoaie@gmail.com

* Correspondence: irina.volf@academic.tuiasi.ro (I.V.); vdinu@icmpp.ro (M.V.D.)

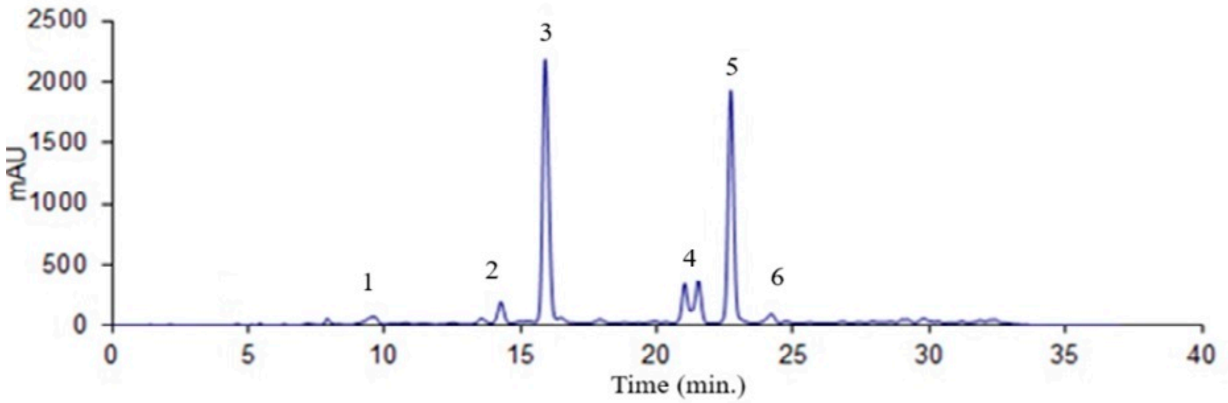

Figure S1. The chromatographic profile of SBE

Table S1. HPLC analysis of SBE

Nr. Crt.	Polyphenolic compound	Chemical formula	Retention time (min)	Peak area	C%	FWHM
1	Catechin	$C_{15}H_{14}O_6$	9.55	42.48	3.21	0.54
2	Vanillic acid	$C_8H_8O_4$	14.24	60.84	4.60	0.25
3	Syringic acid	$C_9H_{10}O_5$	15.90	578.37	43.78	0.24
4	Sinapic acid	$C_{11}H_{12}O_5$	21.50	108.45	8.21	0.25
5	Ferulic acid	$C_{10}H_{10}O_4$	22.72	484.80	36.70	0.22
6	p-Coumaric acid	$C_9H_8O_3$	24.22	45.92	3.47	0.37

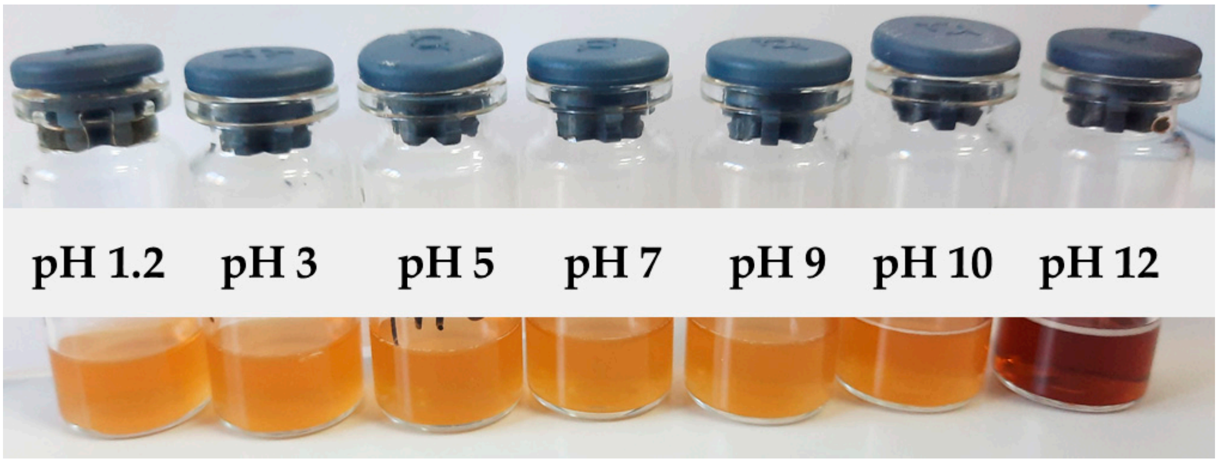

Figure S2. Optical images of SBE in solutions with pH ranging from 1.2 to 12 show no visible changes in appearance up to pH 12. At pH 12, the SBE solution darkens, indicating the oxidation of polyphenolic compounds present in the SBE.

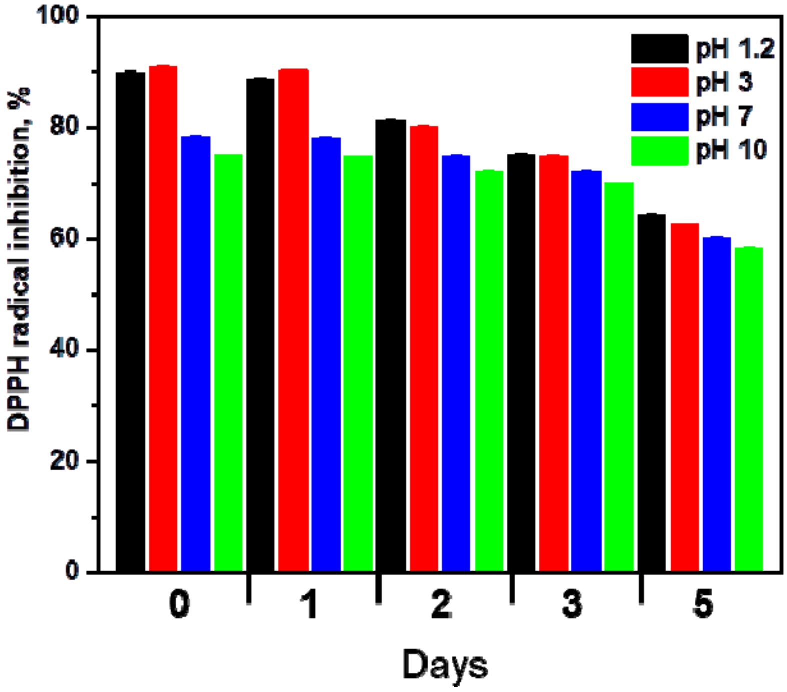

Figure S3. DPPH radical scavenging activity of hydroalcoholic extract from Picea abies bark in solutions with pH values ranging from 1.2 to 10 over 5 days

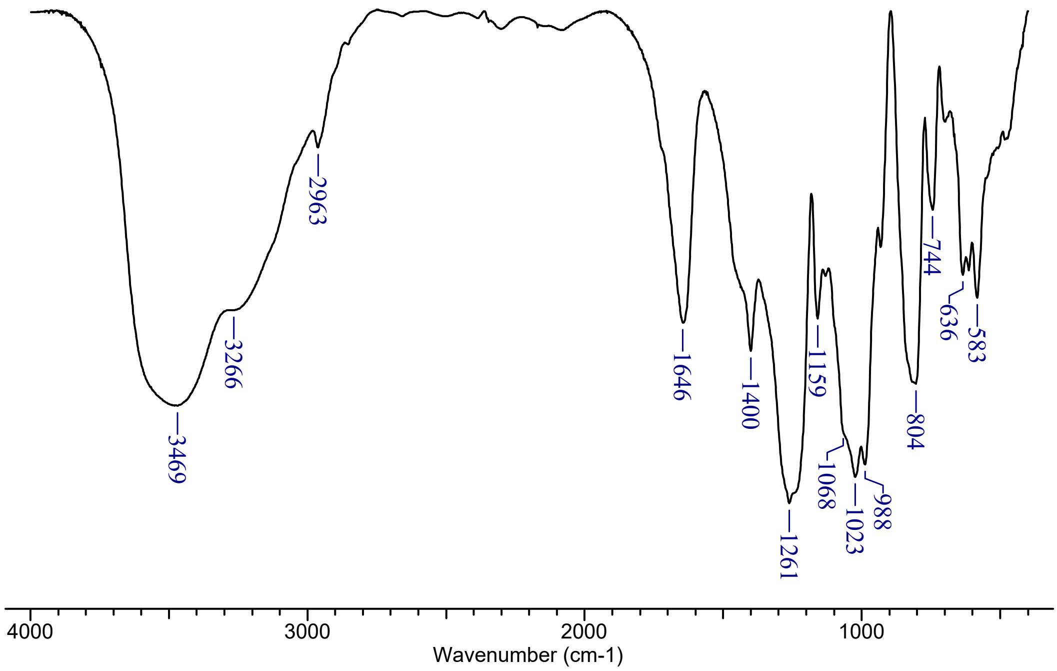

Figure S4. FT-IR spectrum of Dx powder.

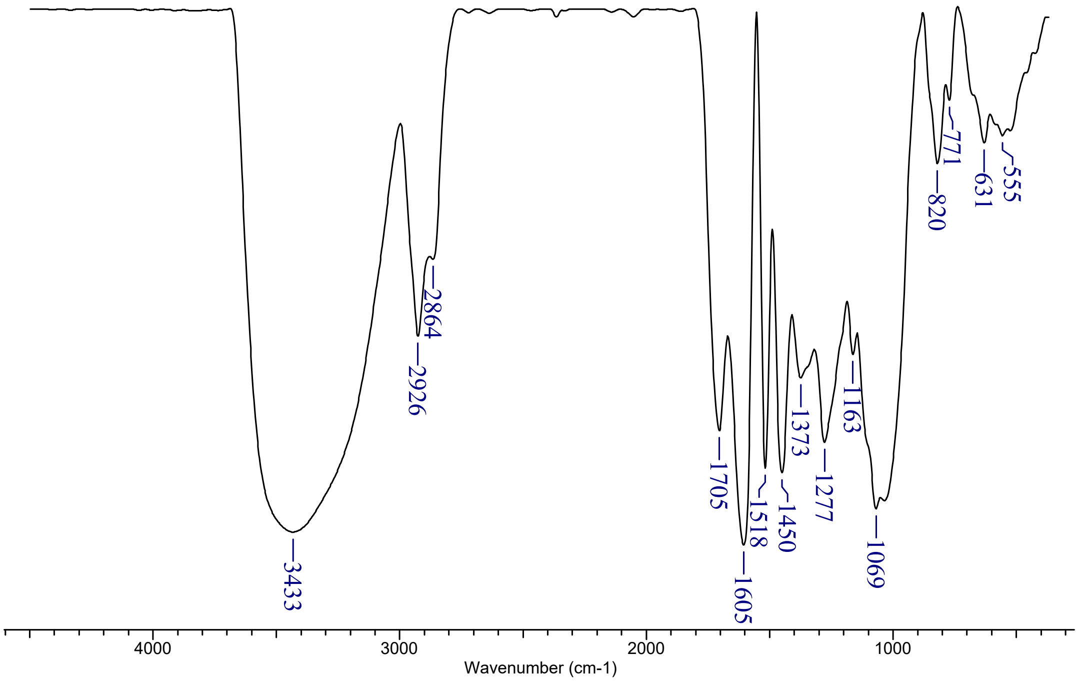

Figure S5. FT-IR spectrum of SBE.

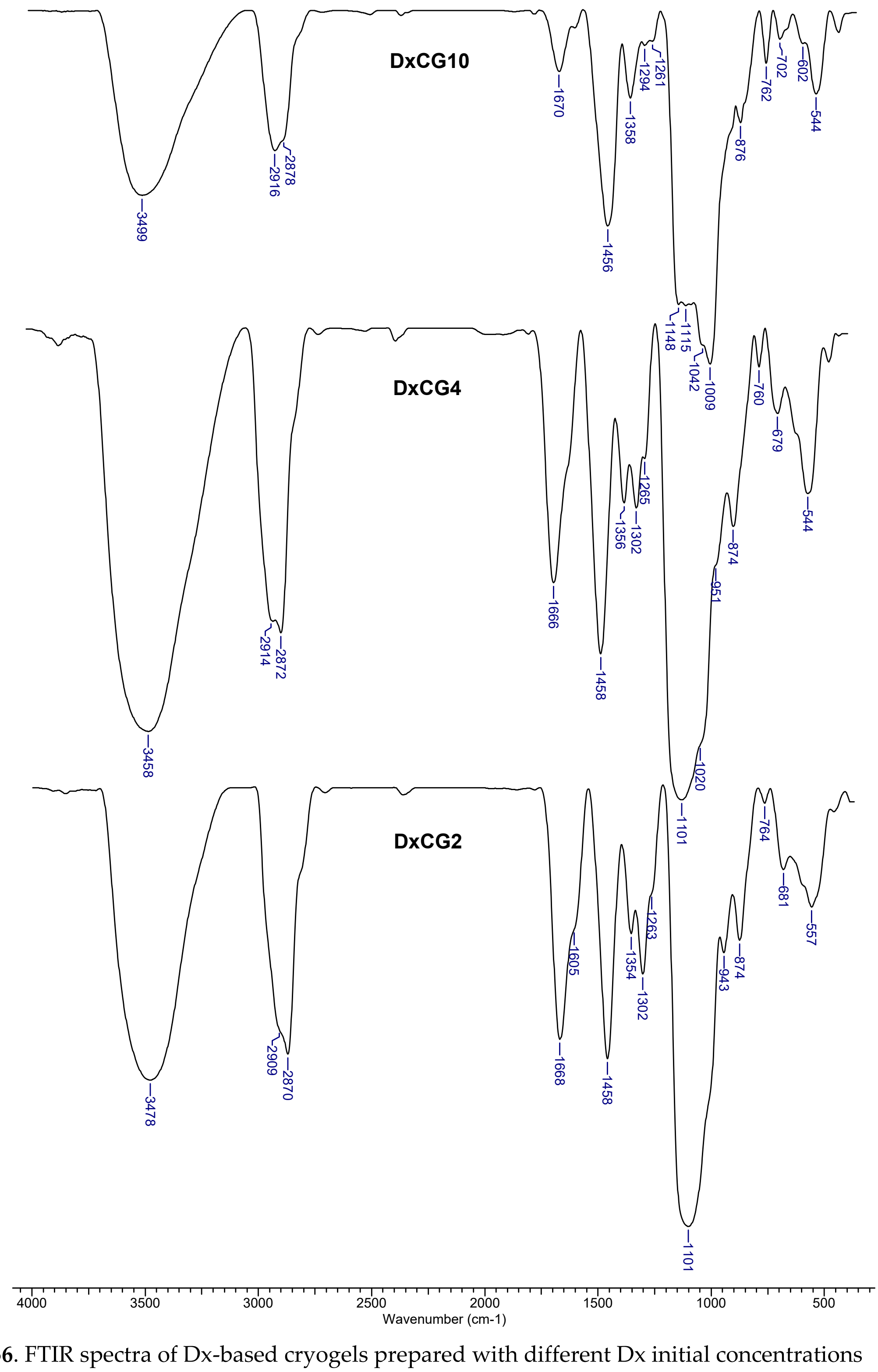

Figure S6. FTIR spectra of Dx-based cryogels prepared with different Dx initial concentrations

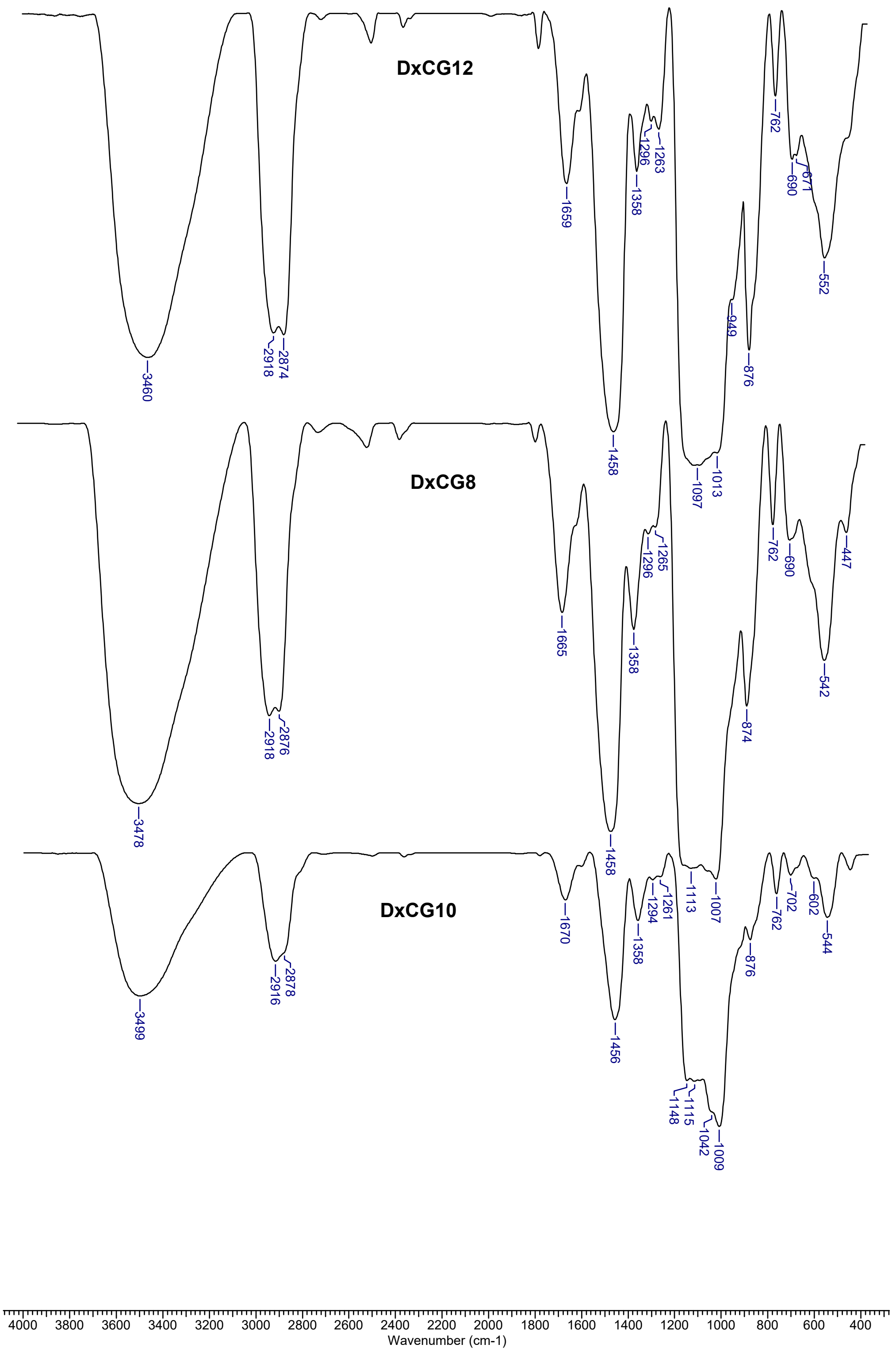

Figure S7. FTIR spectra of Dx-based cryogels prepared with different cross-linker amounts.

Table S2. Weight percentages (wt.%) of elements found on the surface of Dx-based cryogels. Values reported are the average of two measurements.

Elements	DxCG without SBE	DxCG2	DxCG4	DxCG7	DxCG8	DxCG10	DxCG12
	**Wt.%**	**Wt.%**	**Wt.%**	**Wt.%**	**Wt.%**	**Wt.%**	**Wt.%**
C	21.03	44.41	21.85	32.02	38.12	49.26	39.20
N	0.00	0.00	0.00	0.00	0.00	0.00	0.00
O	48.03	20.35	44.65	16.11	15.13	34.29	16.91
Na	30.94	6.91	30.42	22.96	19.15	8.57	17.37
Mg	-	0.05	0.06	0.08	0.03	0.05	0.03
P	-	0.17	0.07	0.06	0.03	0.20	0.03
K	-	0.44	0.14	0.06	0.05	0.54	0.55
Cl	-	27.67	2.82	28.71	27.49	7.09	25.91

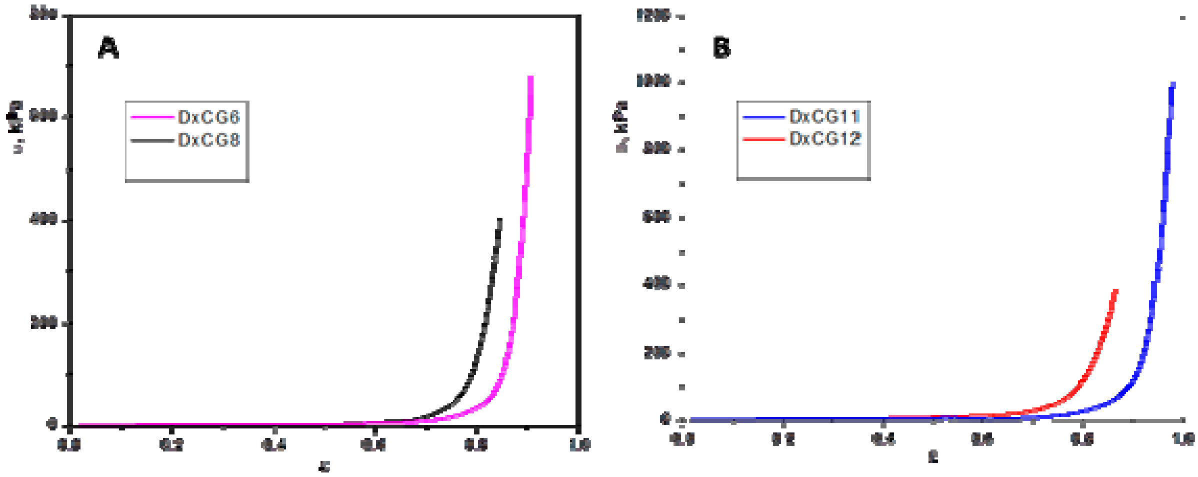

Figure S8. Representative stress-strain (σ-ε) profiles for the DxCG cryogels without (DxCG6 and DxCG11) and with SBE (DxCG8 and DxCG12) obtained by applying a force of 100 N under a displacement control rate of 1 mm min^{-1}.
